# Diagnostic and prognostic biomarker potential of kallikrein family genes in different cancer types

**DOI:** 10.18632/oncotarget.24947

**Published:** 2018-04-03

**Authors:** Prashant D. Tailor, Sai Karthik Kodeboyina, Shan Bai, Nikhil Patel, Shruti Sharma, Akshay Ratnani, John A. Copland, Jin-Xiong She, Ashok Sharma

**Affiliations:** ^1^ Medical College of Georgia, Augusta University, Augusta, GA, USA; ^2^ Center for Biotechnology and Genomic Medicine, Augusta University, Augusta, GA, USA; ^3^ Department of Population Health Sciences, Augusta University, Augusta, GA, USA; ^4^ Department of Pathology, Augusta University, Augusta, GA, USA; ^5^ Mayo Clinic, Jacksonville, FL, USA

**Keywords:** cancer, TCGA, kallikreins, gene expression, prognosis

## Abstract

**Purpose:**

The aim of this study was to compare and contrast the expression of all members of the Kallikrein (KLK) family of genes across 15 cancer types and to evaluate their utility as diagnostic and prognostic biomarkers.

**Results:**

Severe alterations were found in the expression of different Kallikrein genes across various cancers. Interestingly, renal clear cell and papillary carcinomas have similar kallikrein expression profiles, whereas, chromophobe renal cell carcinoma has a unique expression profile. Several KLK genes have excellent biomarker potential (AUC > 0.90) for chromophobe renal cell carcinoma (*KLK2, KLK3, KLK4, KLK7, KLK15*), renal papillary carcinoma (*KLK1, KLK6, KLK7*), clear cell renal cell carcinoma (*KLK1*, *KLK6*), thyroid carcinoma (*KLK2, KLK4, KLK13, KLK15*) and colon adenocarcinoma (*KLK6, KLK7, KLK8, KLK10*). Several KLK genes were significantly associated with mortality in clear cell renal cell carcinoma (*KLK2*: HR = 1.69; *KLK4*: HR = 1.63; *KLK8*: HR = 1.71; *KLK10*: HR = 2.12; *KLK11*: HR = 1.76; *KLK14*: HR = 1.86), papillary renal cell carcinoma (*KLK6*: HR = 3.38, *KLK7*: HR = 2.50), urothelial bladder carcinoma (*KLK5*: HR = 1.89, *KLK6*: HR = 1.71, *KLK8*: HR = 1.60), and hepatocellular carcinoma (*KLK13*: HR = 1.75).

**Methods:**

The RNA-seq gene expression data were downloaded from The Cancer Genome Atlas (TCGA). Statistical analyses, including differential expression analysis, receiver operating characteristic curves and survival analysis (Cox proportional-hazards regression models) were performed.

**Conclusions:**

A comprehensive analysis revealed the changes in the expression of different KLK genes associated with specific cancers and highlighted their potential as a diagnostic and prognostic tool.

## INTRODUCTION

The human kallikrein (KLK) gene family is the largest contiguous cluster of serine proteases within the human genome [1]. The 15 members of the kallikrein gene family are located on chromosome 19q13.4 [2]. *KLK3,* or prostate-specific antigen (PSA), is the most recognized member of the family with its ubiquitous use in prostate cancer screening [1]. Kallikreins are known to play a role in several physiological processes including, extracellular matrix (ECM) remodeling, cellular proliferation, angiogenesis, differentiation, apoptosis, digestive system enzyme activation and coagulation-fibrinolysis [3–5]. Members of the kallikrein family are often expressed in a tissue-specific manner and are regulated by transcriptional and post-transcriptional mechanisms such as steroid hormones (androgen response elements) and serpins, respectively [5–8]. Like other proteases, kallikreins are first secreted as zymogens in both intra and extracellular environments and are activated via other serine proteases or auto-activation [6, 9]. Kallikreins are inactivated by internal cleavage, alpha2-macroglobulin and serpins to prevent excessive proteolytic activity [5, 6]. There is huge variability in the expression of each kallikrein in different tissue types throughout the body [5, 6, 10].

Kallikreins have also been implicated in regulation of tumor growth, neoplastic progression, tumor angiogenesis and metastasis [1, 5, 9, 11–25]. For example, *KLK1*, *KLK2* and *KLK3* can increase degradation of the ECM with their proteolytic effects on fibronectin, laminin, gelatin, fibrinogen and collagenases leading to metastasis [5, 11–16]. In addition, *KLK1-3* have been shown to degrade IGF-binding proteins, releasing free IGF, which increases tumor cell proliferation and survival [5, 14–16]. *KLK4* has been shown to increase the activation of plasmin via activation of urokinase plasminogen activator (uPA) which helps with angiogenesis, invasion, and metastasis of the tumor [5, 17]. *KLK6* and 7 have been implicated in cancer angiogenesis and metastasis by its proteolytic effects cleaving fibrinogen, collagen and laminin [5, 18]. *KLK13* has been shown to have anti-angiogenic properties by creating angiostatin-like fragments, which inhibit angiogenesis [5, 19]. Finally, the kallikreins can activate one another, and cross-activation of kallikreins may be related to malignancies [5, 6, 20, 21].

Kallikreins have shown promise as diagnostic biomarkers, including *KLK3,* which is widely used for screening of prostate cancer [6, 12] and have been associated with prognosis and mortality [21–23]. For example, *KLK15* expression is an independent marker of prognosis in ovarian cancer [2], *KLK9* has been shown to be a marker for prognosis in breast and ovarian cancer [1, 5, 6, 21], and increased *KLK11* expression in gastric carcinoma is associated with poor prognosis [24].

Since different kallikreins have huge variations in expression and function across different tissues, a comprehensive analysis was performed to examine the expression of all members of the kallikrein family genes in different cancer types using The Cancer Genome Atlas (TCGA) gene expression data. The main objectives of this study were to (i) compare and contrast expression of Kallikrein genes across 15 different cancers, (ii) elucidate the effect of kallikreins on the cancer prognosis, and (iii) to evaluate the utility of Kallikrein family as cancer biomarkers.

## RESULTS

### KLK expression changes across cancers

**Figure 1:**
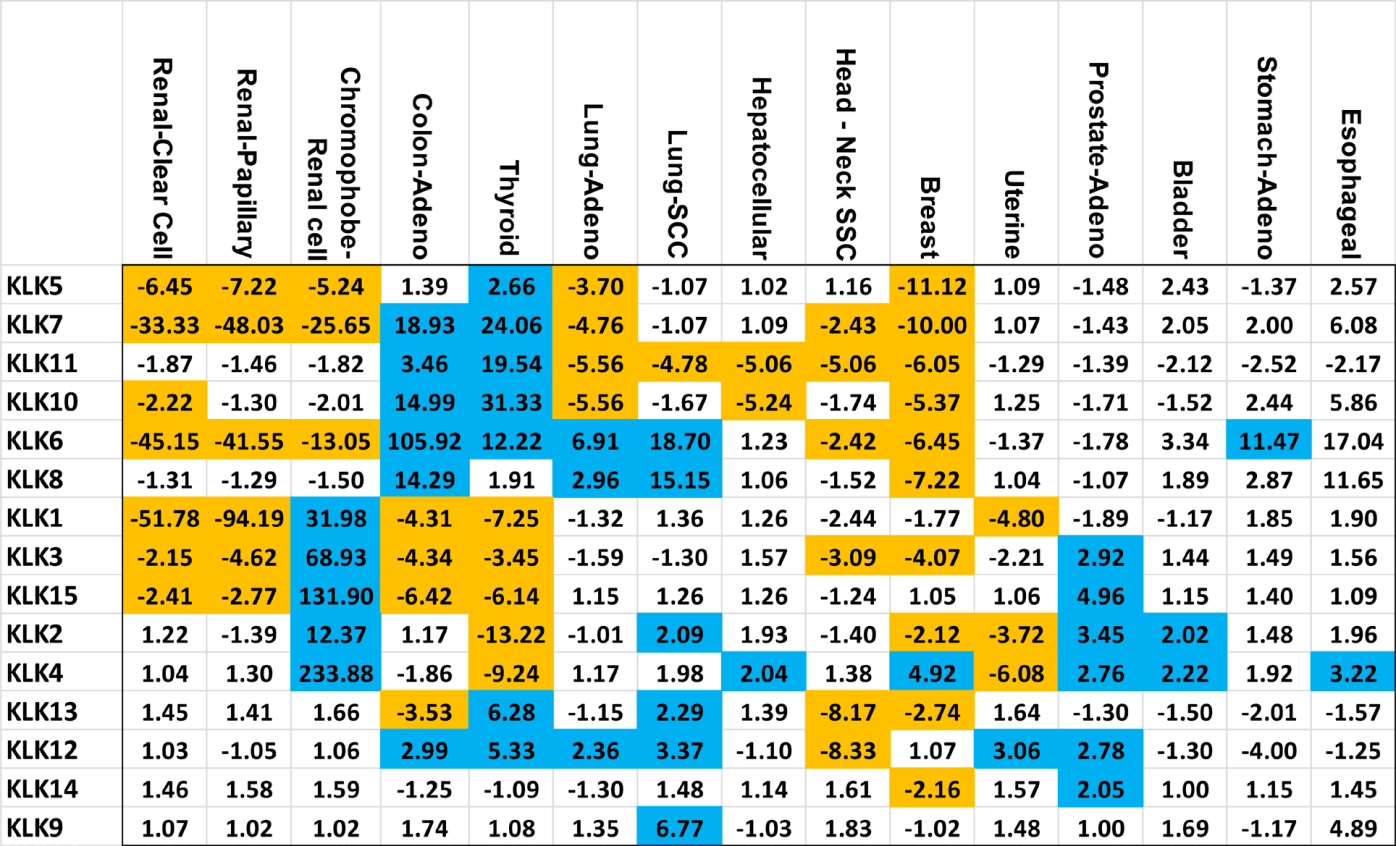
Kallikrein expression fold change in specific cancers Each member of the kallikrein (KLK) family is listed vertically and the 15 cancers are displayed horizontally. Values highlighted in blue or orange represent a fold change greater than 2 or less than 0.5 (–2 fold) respectively while having a *p*-value of less than 0.0033. The vertical and horizontal order was determined by unsupervised clustering of both kallikreins and cancers.

All fifteen members of the kallikrein family (*KLK1-15*) had their expression analyzed in fifteen different cancer types using the gene expression data from TCGA. We have included a table that details the number of tumor and control samples for each cancer type (Table 1). The fold change values comparing tumor and adjacent normal samples are displayed in Figure 1. Bonferroni corrected *p*-value (*p <* 0.0033) was used for statistical significance. All KLKs with at least 2-fold up or down with *p <* 0.0033 are highlighted in Figure 1. Mean expression values in tumor and adjacent normal, fold-changes and *P*-values for each KLK and cancer are included in Supplementary Materials (Supplementary Table 1). The dot-plots showing the distribution of gene expression in cancer patients and adjacent normal for each KLK are shown in Supplementary Materials (Supplementary Figure 1).

**Table 1:** Number of tumor and control samples from 15 cancers analyzed in this study

Cancer type	TCGA code	Tumor samples	Control samples
Breast invasive carcinoma	BRCA	1097	114
Kidney renal clear cell carcinoma	KIRC	533	72
Head and neck squamous cell carcinoma	HNSC	520	44
Lung adenocarcinoma	LUAD	515	59
Thyroid carcinoma	THCA	505	59
Lung squamous cell carcinoma	LUSC	502	51
Prostate adenocarcinoma	PRAD	497	52
Stomach adenocarcinoma	STAD	415	35
Bladder urothelial carcinoma	BLCA	407	19
Liver hepatocellular carcinoma	LIHC	371	50
Kidney renal papillary cell carcinoma	KIRP	290	32
Colon adenocarcinoma	COAD	286	41
Esophageal carcinoma	ESCA	184	11
Uterine corpus endometrial carcinoma	UCEC	176	24
Kidney chromophobe	KICH	66	25

**Figure 2:**
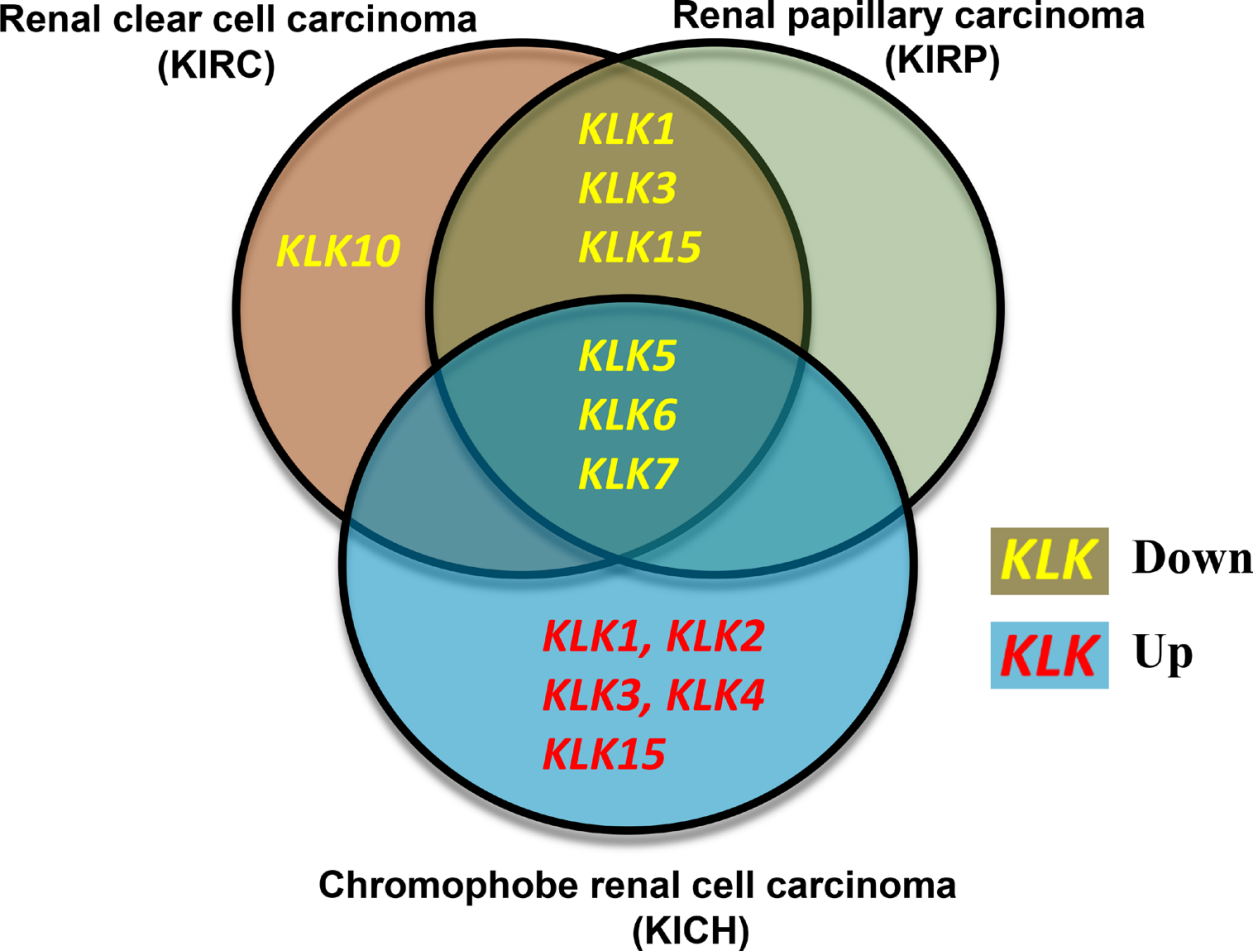
Venn diagram comparing the expression changes of kallikreins in three types of renal cancers Renal clear cell carcinoma and renal papillary carcinoma show similarities in the kallikrein expression changes whereas Chromophobe renal cell carcinoma shows a distinct kallikrein expression profile. Yellow font reflects downregulation whereas red font indicates upregulation of kallikrein expression in carcinoma.

In three types of renal cancers (renal papillary carcinoma, renal clear cell carcinoma and chromophobe renal cell carcinoma), we observed major alterations in the KLK gene expressions. Renal clear cell carcinoma and renal papillary carcinoma solely experienced downregulation of KLK gene expressions; whereas, chromophobe renal cell carcinoma had a combination of increased and decreased expression across the kallikrein family. The renal papillary carcinoma and renal clear cell carcinoma had dramatically decreased expression of *KLK1* (52-fold and 94-fold), *KLK6* (45-fold and 42-fold) and *KLK7* (33-fold and 48-fold), respectively. Chromophobe renal cell carcinoma had increased expression of the following KLKs: *KLK1* (32-fold), *KLK2* (12-fold), *KLK3* (69-fold), *KLK4* (234-fold), *KLK15* (132-fold), whereas decreased expression of *KLK5* (5-fold), *KLK6* (13-fold), and *KLK7* (26-fold). A Venn diagram summarizing our main findings in these three types of renal cancers is shown in Figure 2.

With regards to colon adenocarcinoma and thyroid carcinoma, the following KLKs were significantly elevated: *KLK6* (106-fold and 12-fold), *KLK7* (19-fold and 24-fold), *KLK10* (15-fold and 31-fold), *KLK11* (4-fold and 20-fold), *KLK12* (3-fold and 5-fold), while the following KLKs were significantly downregulated: *KLK1* (4-fold and 7-fold), *KLK3* (4-fold and 3-fold), *KLK15* (6-fold and 6-fold). In addition, thyroid carcinoma displayed significant downregulation of *KLK2* (13-fold) and *KLK4* (9-fold).

Lung adenocarcinoma and lung squamous cell carcinoma showed significant up-regulation of 3 KLKs: *KLK6* (7-fold and 19-fold), *KLK8* (3-fold and 15-fold) and *KLK12* (2-fold and 3-fold) and significant down-regulation of *KLK11* (6-fold and 5-fold). Five KLKs were commonly downregulated in head-neck squamous cell carcinoma and breast invasive carcinoma: *KLK3* (3-fold and 4-fold), *KLK6* (2-fold and 6-fold), *KLK7* (2-fold and 10-fold), *KLK11 (*5-fold and 6-fold) and *KLK13* (8-fold and 3-fold). Prostate adenocarcinoma and urothelial bladder carcinoma exhibited significant up-regulation of *KLK2* and *KLK4* gene expressions (*KLK2:* 3-fold and 2-fold, *KLK4:* 3-fold and 2-fold). Furthermore, uterine corpus endometrial carcinoma displayed significant overexpression of *KLK12* (3-fold) and downregulation of *KLK1* (5-fold), *KLK2* (4-fold) and *KLK4* (6-fold). In each of the following cancers, only one KLK was significantly upregulated: hepatocellular carcinoma (*KLK4*: 2-fold), stomach adenocarcinoma (*KLK6*: 11-fold) and esophageal carcinoma (*KLK4:* 3-fold). The summary of these changes in the expression of KLKs in different cancers is presented in Figure 1.

### Viability of KLKs as cancer biomarkers

**Figure 3:**
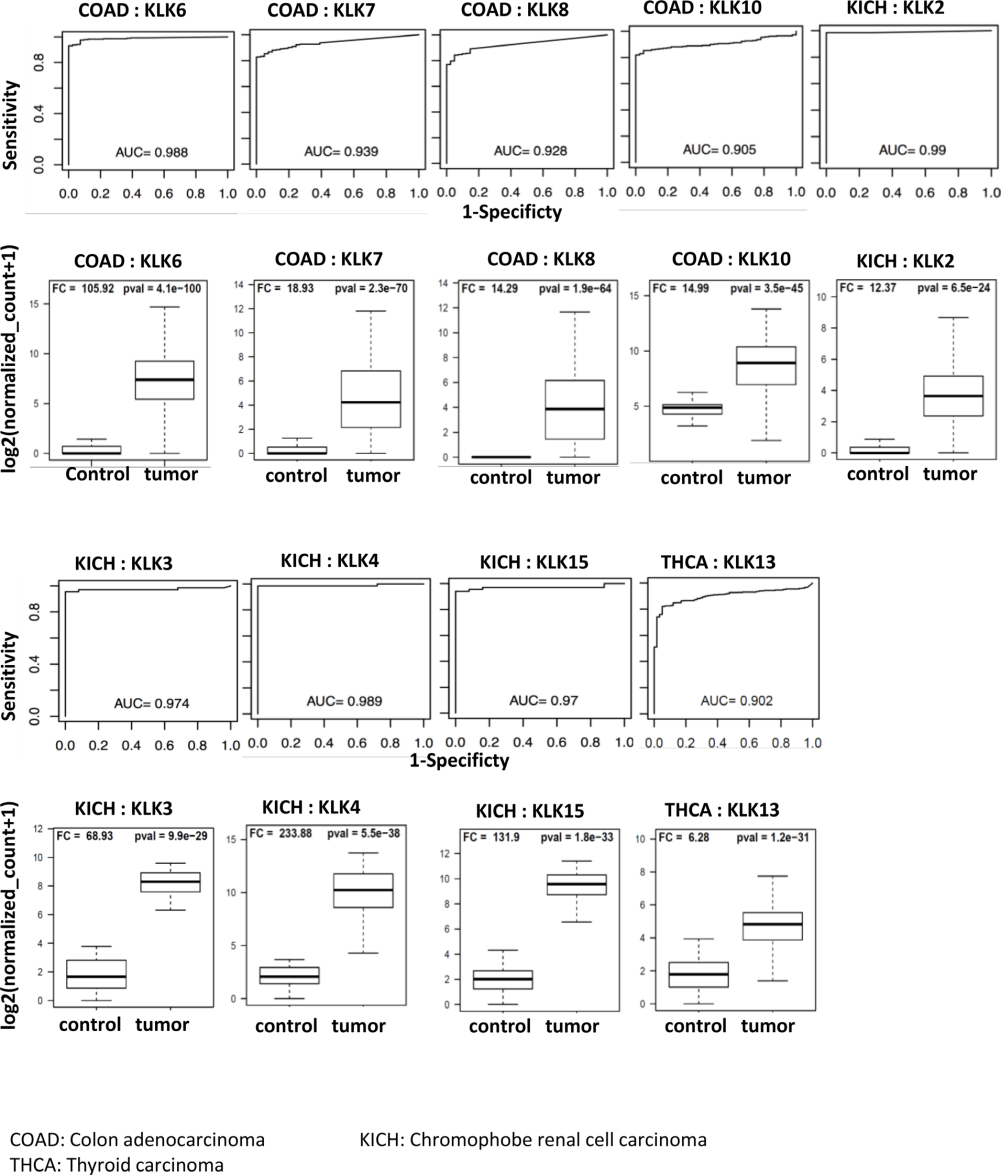
Receiver operating curves (ROC) of kallikreins upregulated in cancers The diagnostic power of individual KLK genes to differentiate cancer patients and respective controls was assessed using the area under the curve (AUC) of the receiver operating characteristic (ROC) curves. The ROC curves and AUC values of top performing KLKs (AUC > 0.90) are shown. The boxplots represent the distribution of expression in tumor and control samples.

To evaluate the biomarker potential of KLKs in 15 cancers, we computed the area under the curve (AUC) of receiver operating characteristic (ROC) curves. The AUC values of all KLKs in 15 cancers are listed in Supplementary Materials (Supplementary Table 1). KLK genes with excellent biomarker potential in different cancers (>5 fold up or down and AUC > 0.90) are shown in Table 2. The ROC curves of up-regulated KLKs with AUC > 0.90 and boxplots depicting their distribution in control and tumor samples are shown in Figure 3. Colon cancer had four up-regulated KLKs with excellent AUC values (*KLK6*: 0.988, *KLK7*: 0.939, *KLK8*: 0.928, *KLK10*: 0.905). Chromophobe renal cell carcinoma also had 4 up-regulated KLKs with excellent diagnostic power (*KLK2*: 0.990, *KLK3*: 0.974, *KLK4*: 0.989, *KLK15*: 0.97). *KLK13* has an AUC value of 0.902 for thyroid carcinoma.

**Table 2:** KLK genes with excellent biomarker potential in 15 different cancer types

Cancer	Gene	Fold change	*P*-value	Normal mean ± SD	Tumor mean ± SD	AUC (95% CI)
**Up-regulated genes**						
COAD	KLK6	105.9	4.14E-100	1.40 ± 1.57	148.76 ± 7.28	0.988 (0.983–0.993)
COAD	KLK7	18.9	2.29E-70	1.21 ± 1.30	22.81 ± 7.69	0.939 (0.926–0.952)
COAD	KLK8	14.3	1.94E-64	1.08 ± 1.23	15.48 ± 7.41	0.928 (0.914–0.942)
COAD	KLK10	15.0	3.52E-45	25.53 ± 1.80	382.65 ± 6.09	0.905 (0.888–0.922)
KICH	KLK2	12.4	6.54E-24	1.11 ± 1.21	13.73 ± 3.75	0.990 (0.981–0.999)
KICH	KLK3	68.9	9.91E-29	3.49 ± 2.23	240.58 ± 3.28	0.974 (0.960–0.988)
KICH	KLK4	233.9	5.51E-38	4.37 ± 2.03	1021.30 ± 4.94	0.989 (0.980–0.998)
KICH	KLK15	131.9	1.81E-33	4.05 ± 2.16	534.27 ± 4.51	0.970 (0.955–0.985)
THCA	KLK13	6.3	1.19E-31	3.57 ± 2.03	22.41 ± 3.01	0.902 (0.888–0.916)
**Down-regulated genes**						
KICH	KLK7	−25.7	2.96E-07	34.89 ± 10.15	1.36 ± 1.56	0.910 (0.871–0.949)
KIRC	KLK1	−51.8	4.34E-24	157.70 ± 9.57	3.05 ± 6.51	0.921 (0.900–0.942)
KIRC	KLK6	−45.2	6.02E-25	96.96 ± 8.02	2.15 ± 3.29	0.928 (0.908–0.948)
KIRP	KLK1	−94.2	2.70E-14	357.84 ± 7.72	3.80 ± 5.92	0.943 (0.915–0.971)
KIRP	KLK6	−41.6	7.81E-13	157.05 ± 6.67	3.78 ± 6.23	0.902 (0.867–0.937)
KIRP	KLK7	−48.0	8.28E-12	86.20 ± 8.12	1.79 ± 2.71	0.906 (0.871–0.941)
THCA	KLK15	−6.1	5.04E-34	16.04 ± 1.95	2.61 ± 3.41	0.905 (0.879–0.931)
THCA	KLK2	−13.2	2.91E-61	124.18 ± 1.77	9.39 ± 4.30	0.943 (0.923–0.963)
THCA	KLK4	−9.2	1.72E-45	103.67 ± 1.91	11.22 ± 4.75	0.914 (0.890–0.938)

**Figure 4:**
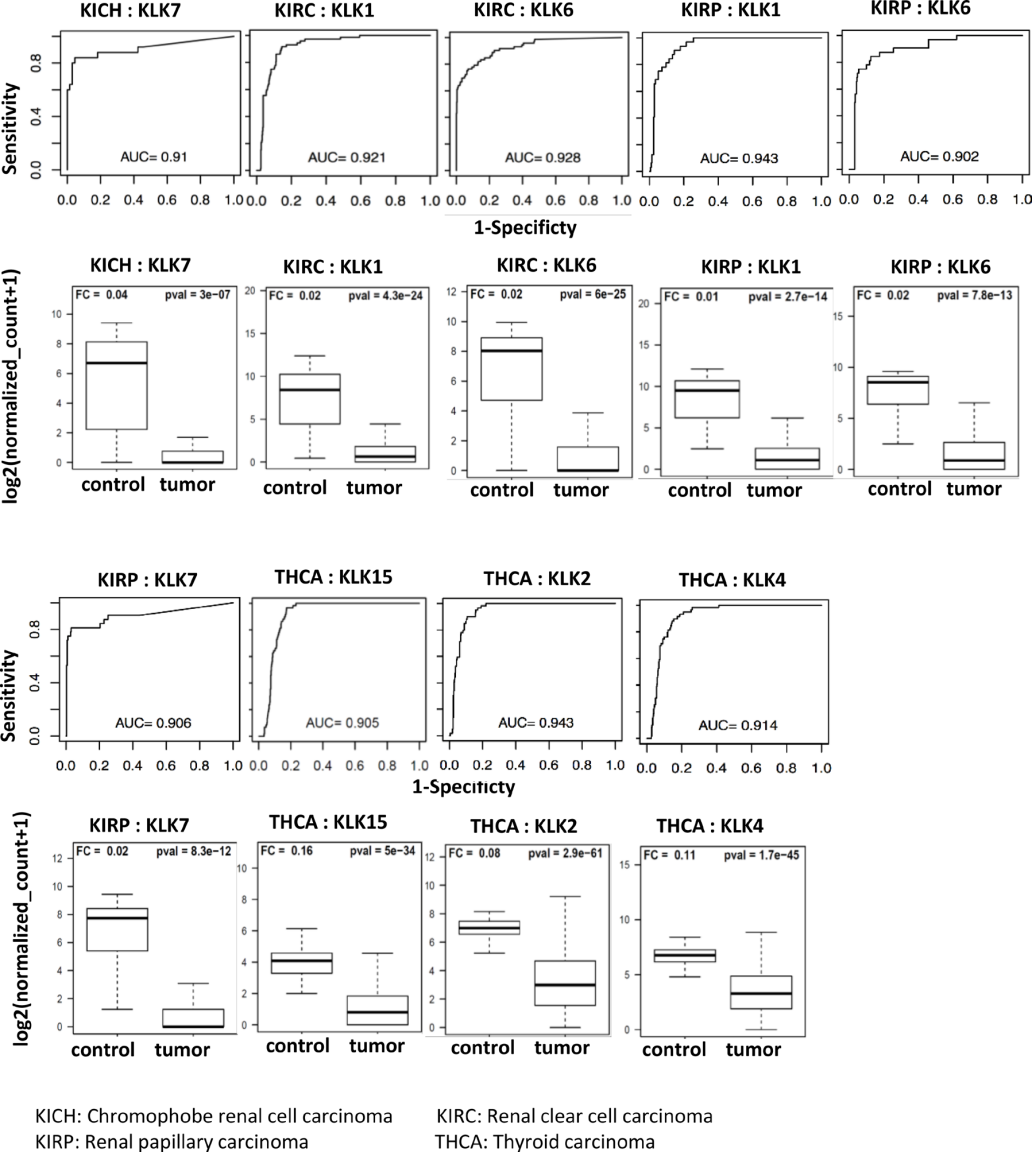
Receiver operating curves (ROC) of kallikreins downregulated in cancers The diagnostic power of individual KLK genes to differentiate cancer patients and respective controls was assessed using the area under the curve (AUC) of the receiver operating characteristic (ROC) curves. The ROC curves and AUC values of top performing KLKs (AUC > 0.90) are shown. Expression of these KLKs is negligible in tumor samples as compared to controls. The boxplots represent the distribution of expression in tumor and control samples.

We also found few downregulated KLKs with AUC > 0.90 as shown in Figure 4. Renal papillary cancer had three KLKs with an AUC of at least 0.90 (*KLK1*: 0.943, *KLK6*: 0.902, *KLK7*: 0.906). Renal clear cell carcinoma had two KLKs with classification power above the AUC threshold (*KLK1*: 0.921, *KLK6*: 0.928). Thyroid carcinoma had three KLKs (*KLK2*: 0.943, *KLK4*: 0.914, *KLK15*: 0.905) that had AUC values above 0.90 threshold and *KLK*7 had an AUC of 0.910 in Chromophobe renal cell carcinoma. These findings are summarized in Table 2.

### KLKs and prognosis relationship across cancers

**Figure 5:**
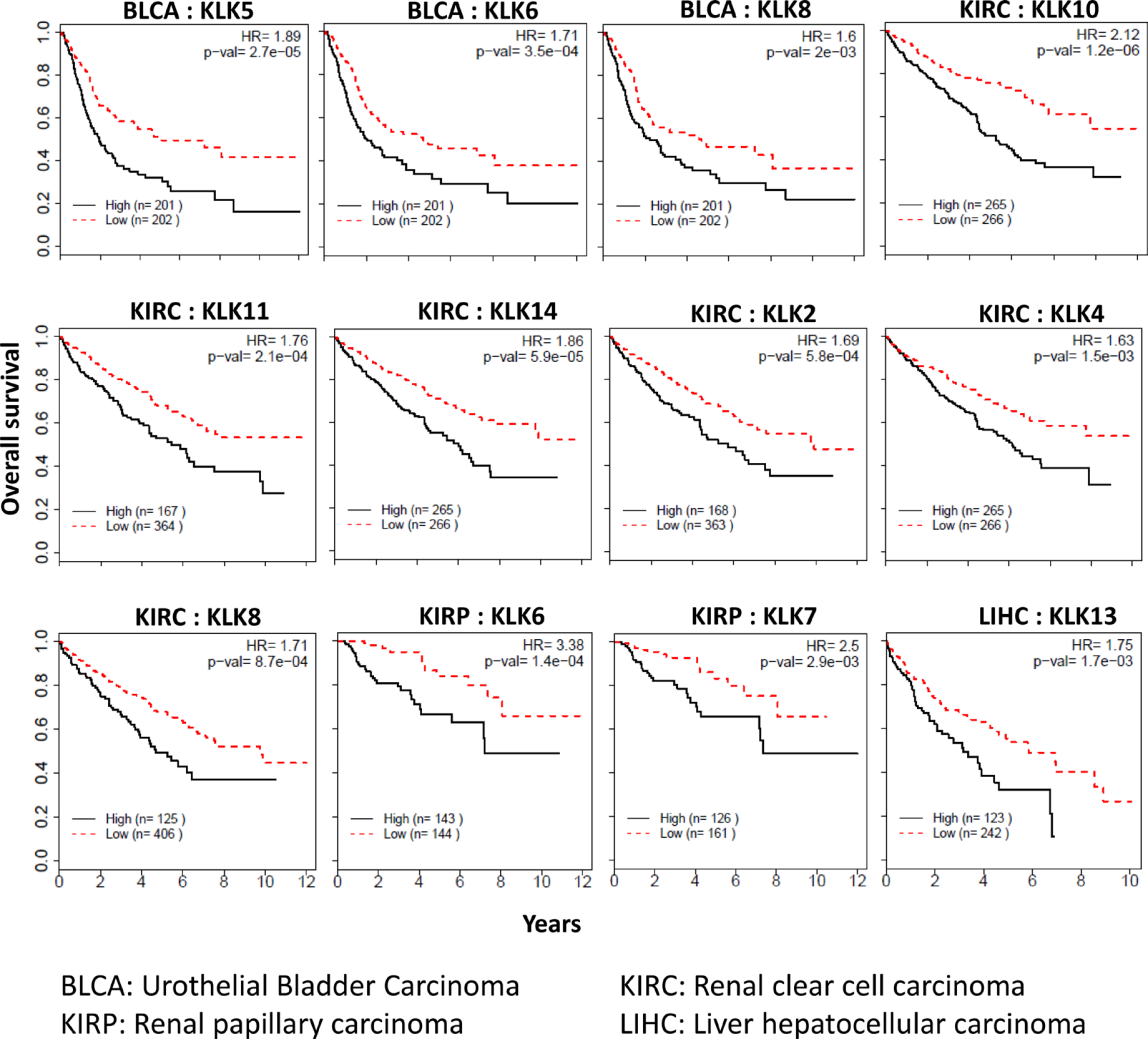
Kallikreins associated with cancer prognosis Kallikreins were independently assessed across the 15 cancers to determine if the expression of kallikrein is associated with overall survival. Kaplan–Meier survival analysis and log-rank test were used to compare differences in overall survival between groups classified using median expression as cut-off. Survival curves, hazard ratio (HR) and *p*-values are shown.

Each of the 15 members of the kallikrein family was independently assessed across the 15 cancers to determine if the expression of kallikrein is associated with overall survival. Prognosis of four (renal clear cell carcinoma, renal papillary carcinoma, urothelial bladder carcinoma, and hepatocellular carcinoma) out of 15 cancers, was found to be significantly associated with kallikrein genes (Figure 5). Renal clear cell carcinoma had six KLKs in which increased expression was associated with overall survival (*KLK2*: HR = 1.69; *KLK4*: HR = 1.63; *KLK8*: HR = 1.71; *KLK10*: HR = 2.12; *KLK11*: HR = 1.76; and *KLK14*: HR = 1.86). In renal papillary carcinoma, increased expression of *KLK*6 (HR = 3.38) and *KLK7* (HR = 2.50) was associated with a significant worsened prognosis. In urothelial bladder carcinoma, increased expression of *KLK5*, *6* and *8* was associated with increased mortality with hazard ratios of 1.89, 1.71, and 1.60, respectively. In hepatocellular carcinoma, increased expression of *KLK13* was significantly associated with increase in mortality (HR = 1.75).

## DISCUSSION

In this study, the expression levels of 15 kallikrein genes were analyzed in 15 cancers using the TCGA gene expression dataset. Apart from changes in the expression, the effect of different KLKs on prognosis of cancers and their biomarker potential was also evaluated. Our study revealed that several KLKs have biomarker potential and can be used clinically in a diagnostic capacity in addition to kallikreins associated with patient survival in four cancer types. Our results support the existing literature that the expression of kallikreins is cancer-specific, as several kallikreins are upregulated in some cancer types and downregulated in other cancers [5]. The most novel findings from this study relate to the renal cell carcinoma subtypes. Our results indicate that renal clear cell and papillary carcinomas have nearly identical decreases in kallikrein expression profiles, whereas chromophobe renal cell carcinoma is a unique subtype as it had dramatic increases in some kallikreins (Figure 2). There is considerable morphology overlap between different subtypes of renal carcinomas [25]. Therefore, the unique association of various kallikreins with these subtypes will provide new biomarkers to differentiate them clinically.

### Renal clear cell carcinoma

All 15 KLKs are known to be expressed in normal kidneys, both at the mRNA and protein level [26, 27]. Renal clear cell carcinoma is the most prevalent subtype of renal cell carcinoma [28]. Our analysis indicated that *KLKs 1*, *3*, *5*, *6*, *7*, *10* and *15* were significantly decreased in renal clear cell carcinoma. The kallikrein-kinin system has been associated with inhibiting various kidney diseases by decreasing reactive oxygen species damage in the kidney; the downregulation of KLKs may decrease these protective effects [29–31]. In an earlier study using immunohistochemical analysis, *KLK6* and *KLK7* have been shown to have lower expression in high grade, in contrast to low grade renal cell carcinoma [32]. Other studies have also shown that expression of several KLKs including *KLKs 1*, *3*, *5*, *6*, *7*, *10* and *15* is either decreased or not detected in renal cancer [27, 33, 34].

### Renal papillary cell carcinoma

In previous studies, renal tubular epithelium has shown cytoplasmic immunohistochemical expression of several KLKs including *KLK5*, *KLK6*, *KLK7*, *KLK10*, *KLK11*, *KLK13* and *KLK14* [34, 35]. Earlier studies indicate decreased expression of *KLKs 1* and *15* and increased expression of *KLKs 6* and *7* in renal papillary cell carcinoma [33, 35, 36]. Our analysis also found significant decreases in the expression of *KLKs 1, 2, 5*, *6, 7* and *15*. The similar profiles in renal clear cell and papillary carcinoma would give credence to the idea that these two cancers might have similar molecular mechanisms. *KLKs 1, 6* and *7* can serve as novel biomarkers for renal papillary carcinoma, where absence of expression of these KLKs indicates tumor. In survival analysis, we confirmed one kallikrein (*KLK6*) that had previously been associated with poor survival [35] and identified one kallikrein (*KLK7*) that previously had not been implicated in prognosis of this cancer.

### Chromophobe renal cell carcinoma

Previously published studies have shown decreased expression of *KLKs 1, 6* and *15* with a weak expression of *KLK7* with immunohistochemical analysis [32]. Our analysis differed and found that *KLK1*, *KLK2*, *KLK3*, *KLK4* and *KLK15* are highly upregulated in chromophobe renal cell carcinoma. We hypothesize that the increase in these five kallikreins may cause increased expression of uPA and the destruction of the ECM, which has been shown to be active in renal carcinoma [37–39]. *KLK5* and *KLK6* are decreased in all three types of the renal carcinomas. We identified four viable cancer biomarkers (*KLK2*, *KLK3*, *KLK4* and *KLK15*) for chromophobe renal cell carcinoma with almost perfect sensitivity and specificity (AUC values: 0.97–0.99).

### Colon adenocarcinoma

Significant increases in expression of *KLKs 6, 7, 8, 10, 11* and *12* and decreases in *KLKs 1, 3, 13* and *15* were found in analysis of colon adenocarcinoma. Previous literature has shown increased expression of *KLKs 6, 7, 10, 11* and *15* [40–46]. The nearly 19-fold increase in *KLK7* is consistent with Walker *et al.*’s findings of increased expression in *KLK7* leading to enhanced cell proliferation in the colon [47]. *KLK7* expression is also increased in the thyroid; however, the majority of the tissues experienced a downregulation of *KLK7*, indicating that the cell proliferative effects might be tissue-specific. The increase in *KLK12* may lead to increased angiogenesis in endothelial cells as described in a prior study [48]. Our analysis indicated that KLKs 6, 7, 8 and 10 are excellent diagnostic biomarkers for colon adenocarcinoma. Talieri *et al.*, found that *KLK5, KLK7 and KLK14* were significant factors for prognosis [49]; however, we did not find any kallikreins significantly associated with prognosis of colon adenocarcinoma.

### Thyroid carcinoma

Thyroid hormones regulate kallikrein levels, and the dysfunction of thyroid hormones during thyroid carcinoma may lead to the dramatic changes in the kallikreins’ expression [19, 50]. Our analysis indicated significant increases in KLKs 5, 6, 7, 10, 11, 12 and 13 and decreases in KLKs 1, 2, 3, 4 and 15. Previous studies have obtained similar results concerning the expression of KLKs 2, 3 and 7 [51, 52]. Also, our ROC analysis indicates that *KLK2* and *KLK4* can be used as novel biomarkers for thyroid cancer, since previous studies have not reported any kallikreins as biomarkers for thyroid cancer.

### Lung adenocarcinoma

Significant decreases in *KLKs 5, 7, 10* and *11*, while significant increases in *KLKs 6, 8* and *12* were observed in differential expression analysis. Previous studies have also found increased expression of *KLKs 5, 10* and *11* and decreased expression of 6, 8, 7, and 12 in lung adenocarcinoma [53–56]. *KLK12* is known to stimulate angiogenesis via a platelet-derived growth factor-B (*PDGFB*) dependent paracrine pathway [48]. Increased *KLK8* has been associated with a favorable clinical outcome in lung adenocarcinoma by suppressing tumor invasiveness through inhibition of integrin signaling and cell adhesion [57]; however we could not confirm this finding in our study.

### Lung squamous cell carcinoma

Differential expression analysis indicated increased expression of KLKs 2, 6, 8, 9, 12 and 13 and decreased expression of *KLK11*. However, *KLK11* is known to be overexpressed lung squamous cell carcinoma [58]. Previous studies have found similar increased expression patterns for *KLK6* and *KLK8* [55, 59]. The increased expression of KLKs 2, 9, 12 and 13 are novel findings in our study.

### Hepatocellular carcinoma

Hepatocellular carcinoma has a significant decrease in the expression of *KLK10* and *KLK11*. Decreased expression of *KLK10* is consistent with earlier findings [44] while the decreased expression of *KLK11* in hepatocellular carcinoma is previously unknown.

### Head-neck squamous cell carcinoma

*KLK6* expression was decreased in head-neck squamous cell carcinoma, in contrast to earlier findings [60]. We also found significant decrease in the expression of *KLKs3*, 7, 11, 12 and 13 that were previously not known. A prior study found increased expression of *KLK10* in Group 1 tumor subtype; however, our analysis did not find *KLK10* expression to be significantly different [61].

### Breast invasive carcinoma

KLKs 2, 3, 5, 6, 7, 10, 11, 13 and 14 were found to have significantly decreased expression in breast invasive carcinoma. These findings are mostly consistent with previously published studies [6, 9, 22, 62–68]. Decreased *KLK7* in breast cancer is a novel finding. *KLK4* was the only kallikrein overexpressed and was consistent with earlier findings of increased expression in malignant tumors [69].

### Uterine corpus endometrial carcinoma

The expression of three kallikreins (*KLKs1*, 2 and 4) was found to be decreased in uterine endometrial cancer. Dorn *et al.* found similar expression changes for *KLK1* but also found increases in the expression of *KLKs6*, 8 and 10 [36].

### Prostate adenocarcinoma

Differential expression analysis indicated dramatic changes in the expression of *KLK 2, 3, 4, 12, 14* and *15*. Published literature is consistent with *KLK3*, *4*, *14* and *15* expression changes, but was the opposite for *KLK2* in malignant tumors [6, 70]. Increased expression of *KLK12* was not reported earlier and serves as a new discovery.

### Urothelial bladder carcinoma

Our analyses found significant increases in the expression of *KLK2* and *KLK4*. We also found that three kallikreins (KLKs 5, 6 and 8) were significantly associated with overall survival in urothelial bladder carcinoma. Previous studies found increased expression in KLKs 5, 6, 8 and 9 [6, 36]. Also, down-regulation of *KLK13* was associated with an unfavorable prognosis [71]; however we did not find KLK13 expression to have a significant effect on survival.

### Stomach adenocarcinoma

In stomach adenocarcinoma, the only significant change in expression was found in *KLK6* (11-fold upregulated). Other studies for gastric cancer have found increased expression of *KLKs6* and 12 and decreased expression of *KLKs10*, 11 and 13 [6, 44, 72].

### Esophageal carcinoma

In our analysis *KLK4* expression was increased 3-fold in esophageal carcinoma. Two earlier studies have found increased expression of *KLK6* [44, 66]. In our study *KLK6* was increased in esophageal carcinoma, but could not achieve statistical significance.

## METHODS

### TCGA datasets

The TCGA gene expression RNAseq data (IlluminaHiSeq: log2-normalized_count+1) was downloaded from Xena browser (https://xenabrowser.net/datapages/). We selected 15 cancer types for analyses having at least 10 adjacent normal samples: Renal clear cell carcinoma (KIRC), Renal papillary carcinoma (KIRP), Chromophobe renal cell carcinoma (KICH), Colon adenocarcinoma (COAD), Thyroid carcinoma (THCA), Lung adenocarcinoma (LUAD), Lung squamous cell carcinoma (LUSC), Hepatocellular carcinoma (LIHC), Head and Neck squamous cell carcinoma (HNSC), Breast invasive carcinoma (BRCA), Uterine Corpus Endometrial Carcinoma (UCEC), Prostate adenocarcinoma (PRAD), Urothelial Bladder Carcinoma (BLCA), Stomach adenocarcinoma (STAD) and Esophageal carcinoma (ESCA). Statistical analyses were performed to compare and contrast the expression levels of 15 kallikrein genes in these 15 cancer types.

### Statistical analyses

All statistical analyses were performed using the R language and environment for statistical computing (R version 3.2.2; R Foundation for Statistical Computing; www.r-project.org). The potential differences in the gene expression between cancer patients and adjacent normal were examined. To express the amount and significance of change, Fold change (FC: Cancer *vs* Adjacent Normal) and *p*-values (pval) were calculated. The dot plots were created to visualize the distribution of gene expression in cancer patients and adjacent normal (expression levels in each subject represented by a dot). The diagnostic power of individual KLK genes to differentiate cancer patients and respective controls was assessed using the area under the curve (AUC) of the receiver operating characteristic (ROC) curves. We used Cox proportional hazards models to evaluate the impact of gene expression levels on overall survival. Overall survival data (diagnosis to date of death) were downloaded from the TCGA patient phenotype files. Patients who are alive with no evidence of disease were censored at the date of last follow-up visit. Kaplan-Meier survival analysis and log-rank test were used to compare differences in overall survival between groups classified using different cut-offs of expression level.

## CONCLUSIONS

Previous studies have shown that kallikreins can serve as biomarkers to help diagnose individuals with cancer and are also associated with prognosis and mortality. However, the expression and function of kallikreins is known to be tissue-specific. Our analysis explored the expression of every member of the kallikrein family in 15 cancer types, yielding a comprehensive overview of KLK expression in these cancers. In this study, differential expression analysis indicated that every cancer had at least one kallikrein that had a significant change in expression, with some cancers having up to 12 kallikreins significantly altered. Analysis of kallikrein expression performed on the subtypes of renal cell carcinoma was essentially novel as the subtypes had been explored minimally in previous studies. Renal clear cell and papillary carcinomas appear to have similar kallikrein expression, while chromophobe renal cell carcinoma displayed dramatic overexpression. Our analysis indicates multiple kallikreins can serve as biomarkers for diagnosis for all three renal subtypes with high sensitivity and specificity. Novel diagnostic biomarkers for colon adenocarcinoma and thyroid carcinoma were also identified. In addition to the renal carcinomas, several kallikreins in urothelial bladder carcinoma and head-neck squamous cell carcinoma can serve as prognostic indicators. Further studies need to be conducted exploring the utilization of kallikreins as a diagnostic or prognostic tool. Also, studies are needed to elucidate the precise mechanisms of kallikreins in various cancers which can help in personalized medicine and lead to improved outcomes in patients with more specific drug regimes.

## SUPPLEMENTARY MATERIALS FIGURE AND TABLE




