# Supplementary material for: Diagnostic and prognostic biomarker potential of kallikrein family genes in different cancer types
Source: Oncotarget. 2018 Apr 3;9(25):17876–88. doi: 10.18632/oncotarget.24947 (PMC5915161; doi:10.18632/oncotarget.24947)
Supplement: Supplementary file 1 [file oncotarget-09-17876-s001.pdf]

Diagnostic and prognostic biomarker potential of kallikrein family genes in different cancer types

SUPPLEMENTARY MATERIALS

Supplementary Table 1: Fold change (FC), AUC values, Hazards ratios and the mean expression values for each KLK in 15 cancers. See Supplementary_Table_1

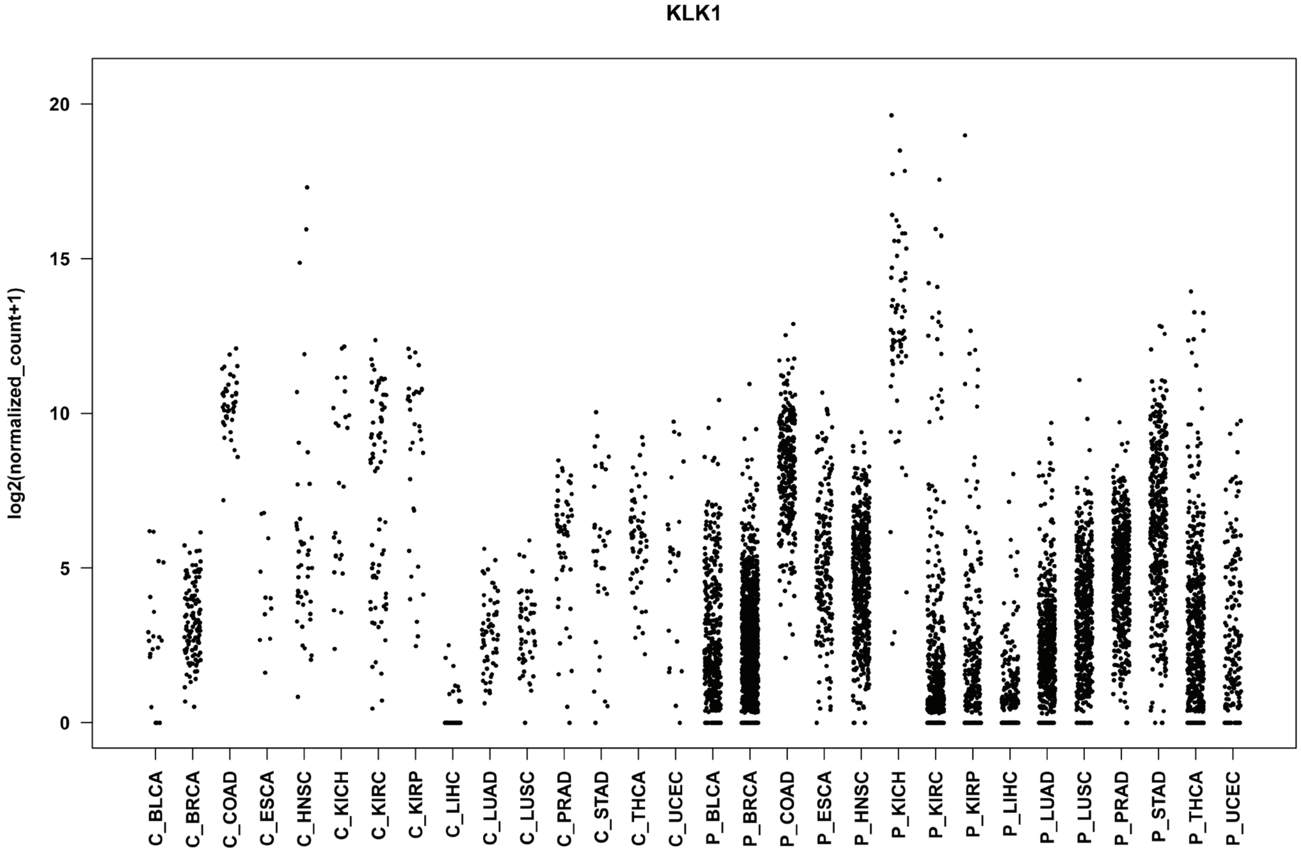

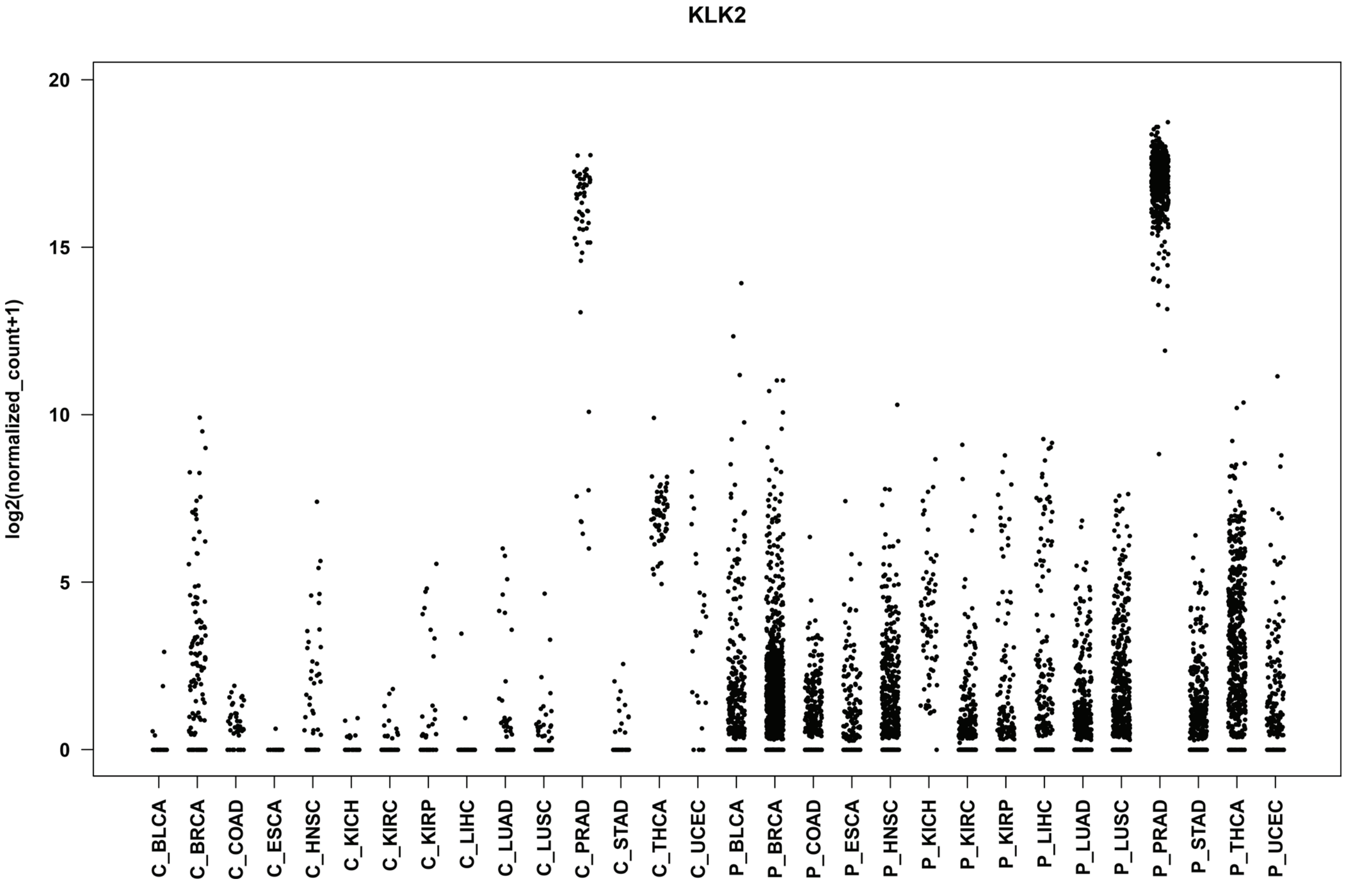
KLK2
log2(normalized_count+1)
0
5
10
15
20
C_BLCA
C_BRCA
C_COAD
C_ESCA
C_HNSC
C_KICH
C_KIRC
C_KIRP
C_LIHC
C_LUAD
C_LUSC
C_PRAD
C_STAD
C_THCA
C_UCEC
P_BLCA
P_BRCA
P_COAD
P_ESCA
P_HNSC
P_KICH
P_KIRC
P_KIRP
P_LIHC
P_LUAD
P_LUSC
P_PRAD
P_STAD
P_THCA
P_UCEC

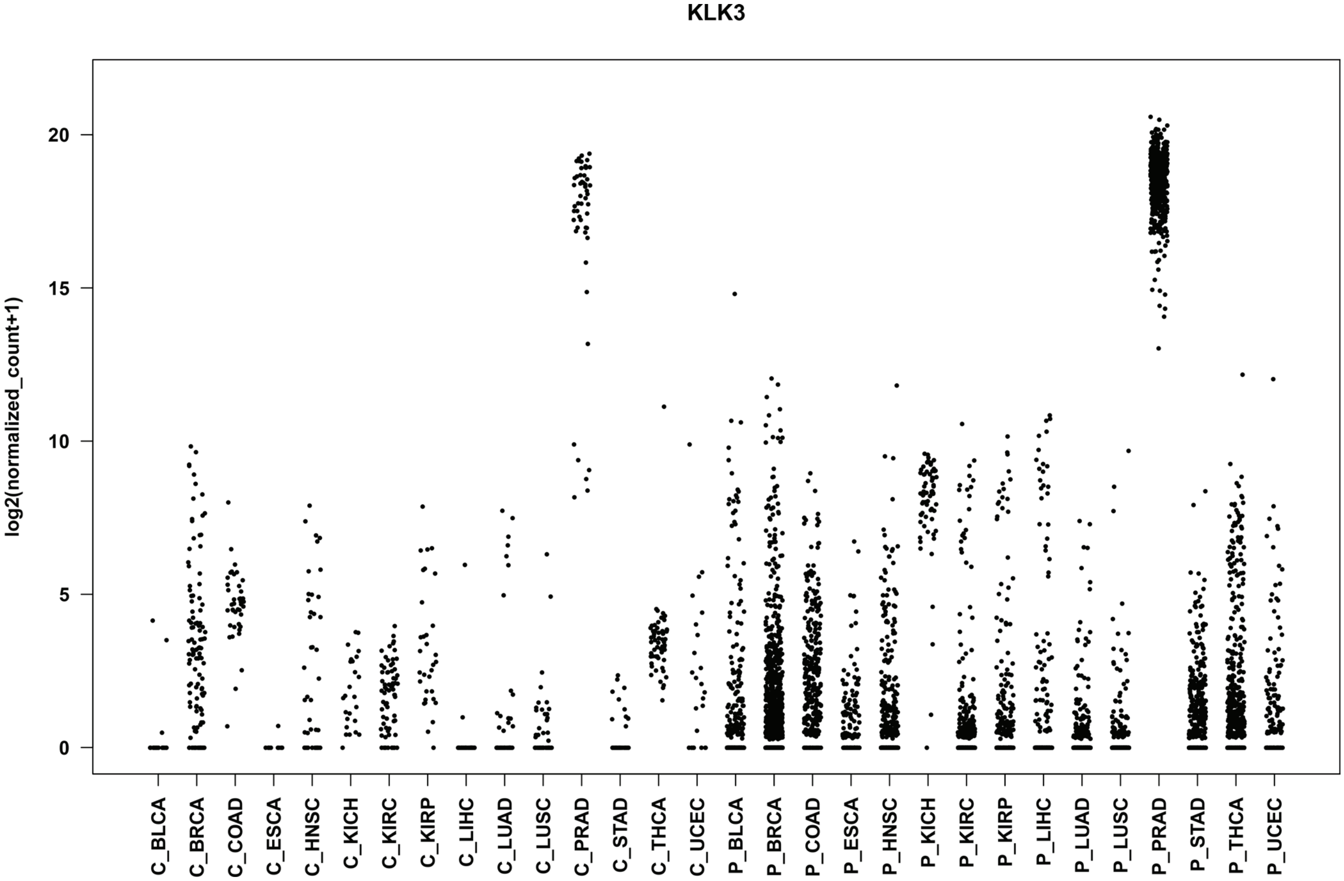
KLK3
log2(normalized_count+1)
0
5
10
15
20
C_BLCA
C_BRCA
C_COAD
C_ESCA
C_HNSC
C_KICH
C_KIRC
C_KIRP
C_LIHC
C_LUAD
C_LUSC
C_PRAD
C_STAD
C_THCA
C_UCEC
P_BLCA
P_BRCA
P_COAD
P_ESCA
P_HNSC
P_KICH
P_KIRC
P_KIRP
P_LIHC
P_LUAD
P_LUSC
P_PRAD
P_STAD
P_THCA
P_UCEC

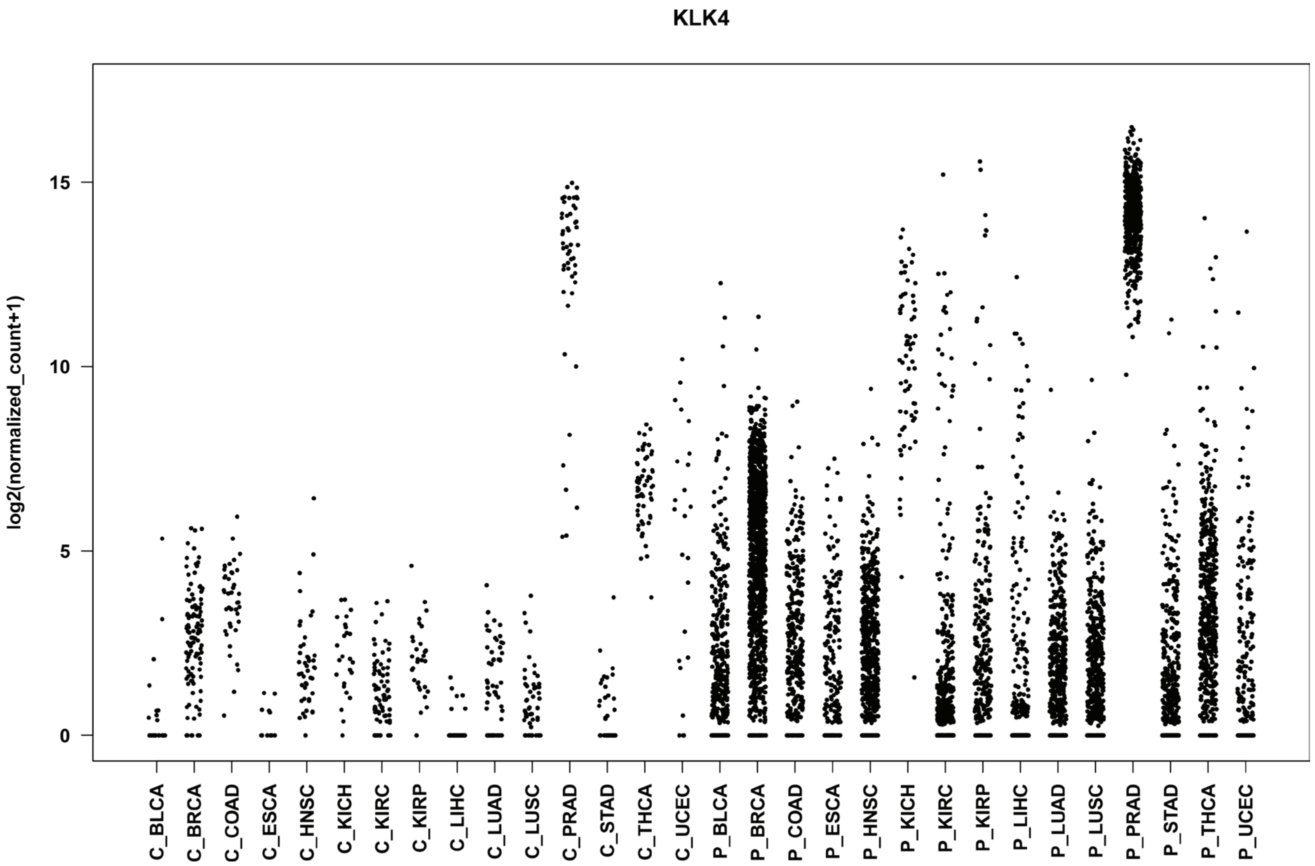
KLK4
log2(normalized_count+1)
0
5
10
15
C_BLCA
C_BRCA
C_COAD
C_ESCA
C_HNSC
C_KICH
C_KIRC
C_KIRP
C_LIHC
C_LUAD
C_LUSC
C_PRAD
C_STAD
C_THCA
C_UCEC
P_BLCA
P_BRCA
P_COAD
P_ESCA
P_HNSC
P_KICH
P_KIRC
P_KIRP
P_LIHC
P_LUAD
P_LUSC
P_PRAD
P_STAD
P_THCA
P_UCEC

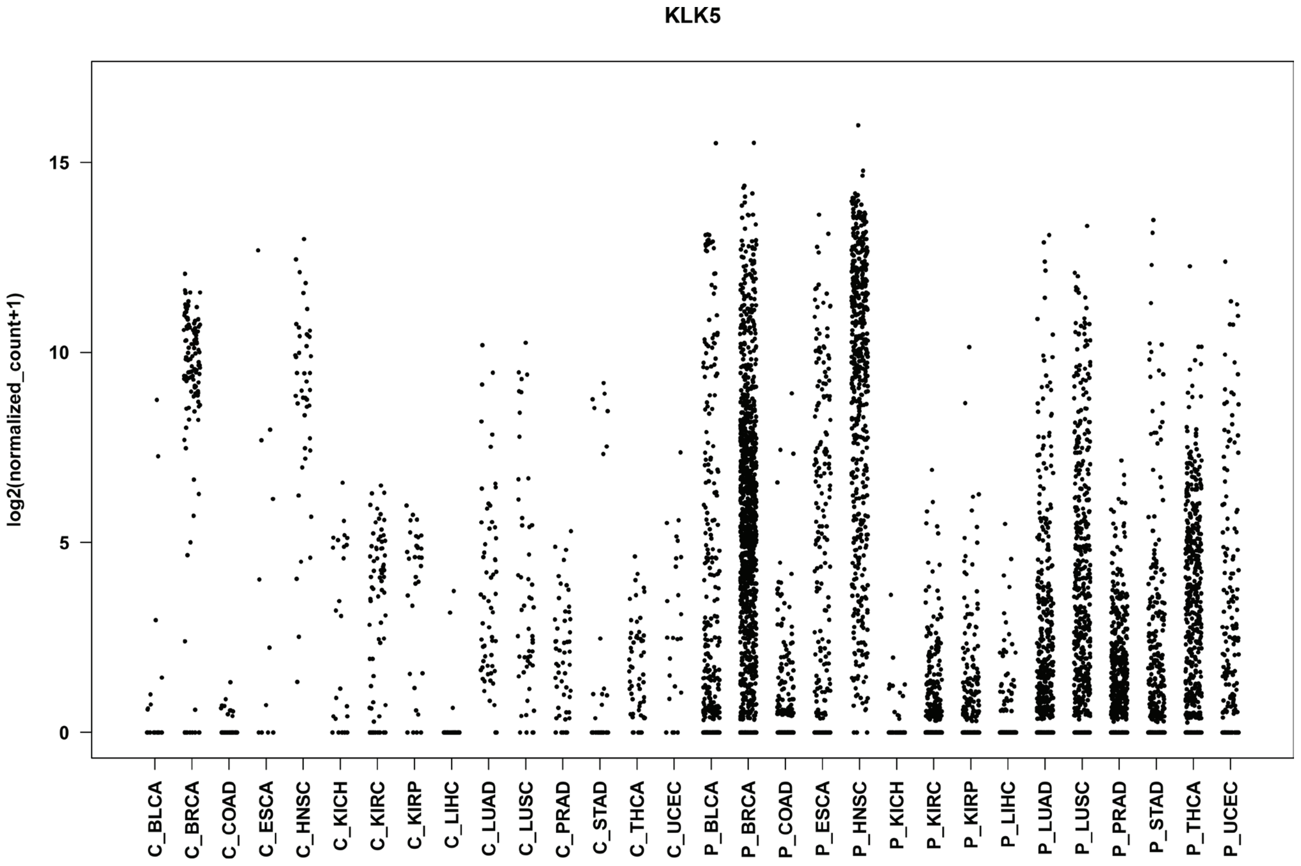
KLK5
log2(normalized_count+1)
0
5
10
15
C_BLCA
C_BRCA
C_COAD
C_ESCA
C_HNSC
C_KICH
C_KIRC
C_KIRP
C_LIHC
C_LUAD
C_LUSC
C_PRAD
C_STAD
C_THCA
C_UCEC
P_BLCA
P_BRCA
P_COAD
P_ESCA
P_HNSC
P_KICH
P_KIRC
P_KIRP
P_LIHC
P_LUAD
P_LUSC
P_PRAD
P_STAD
P_THCA
P_UCEC

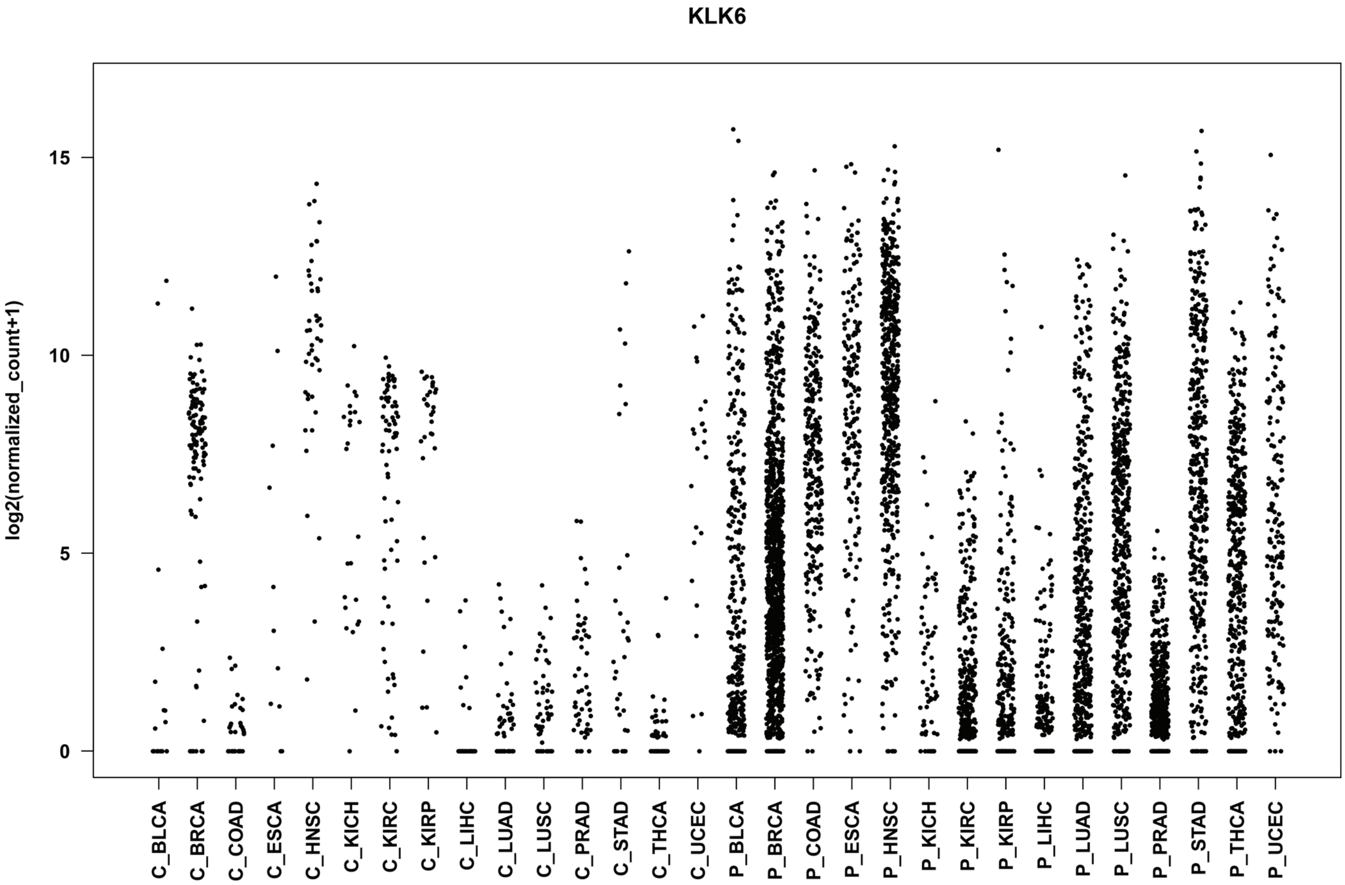
KLK6
log2(normalized_count+1)
0
5
10
15
C_BLCA
C_BRCA
C_COAD
C_ESCA
C_HNSC
C_KICH
C_KIRC
C_KIRP
C_LIHC
C_LUAD
C_LUSC
C_PRAD
C_STAD
C_THCA
C_UCEC
P_BLCA
P_BRCA
P_COAD
P_ESCA
P_HNSC
P_KICH
P_KIRC
P_KIRP
P_LIHC
P_LUAD
P_LUSC
P_PRAD
P_STAD
P_THCA
P_UCEC

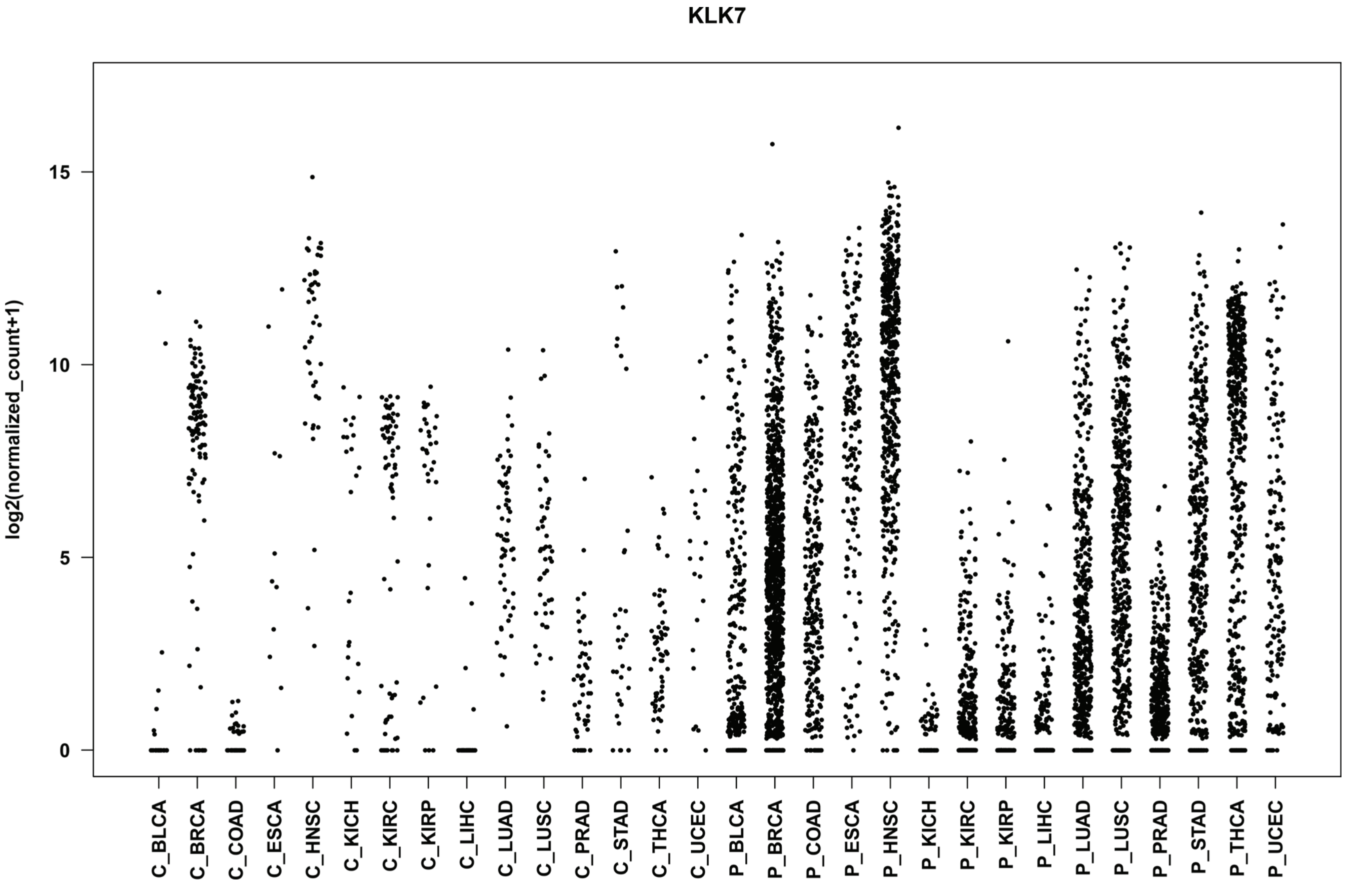
KLK7
log2(normalized_count+1)
0
5
10
15
C_BLCA
C_BRCA
C_COAD
C_ESCA
C_HNSC
C_KICH
C_KIRC
C_KIRP
C_LIHC
C_LUAD
C_LUSC
C_PRAD
C_STAD
C_THCA
C_UCEC
P_BLCA
P_BRCA
P_COAD
P_ESCA
P_HNSC
P_KICH
P_KIRC
P_KIRP
P_LIHC
P_LUAD
P_LUSC
P_PRAD
P_STAD
P_THCA
P_UCEC

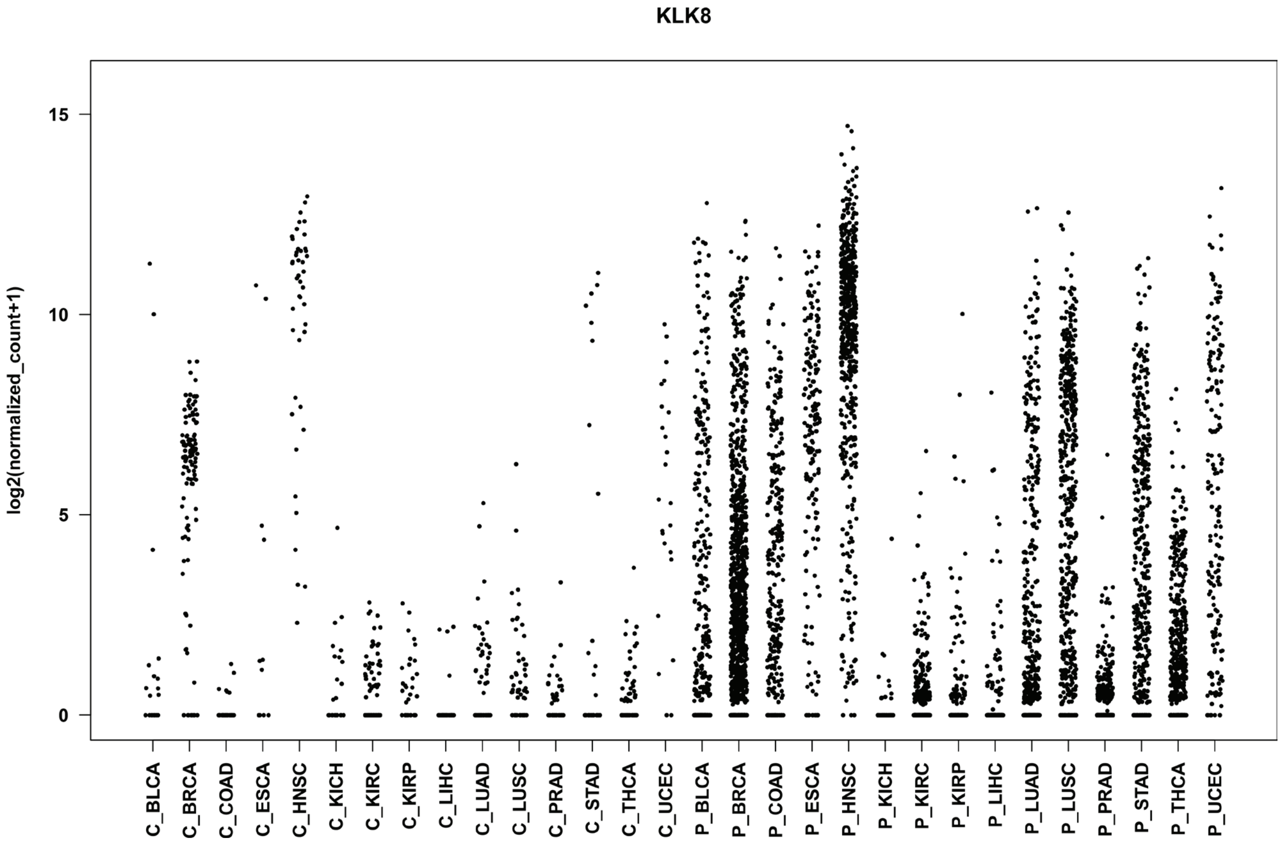
KLK8
log2(normalized_count+1)
15
10
5
0
C_BLCA
C_BRCA
C_COAD
C_ESCA
C_HNSC
C_KICH
C_KIRC
C_KIRP
C_LIHC
C_LUAD
C_LUSC
C_PRAD
C_STAD
C_THCA
C_UCEC
P_BLCA
P_BRCA
P_COAD
P_ESCA
P_HNSC
P_KICH
P_KIRC
P_KIRP
P_LIHC
P_LUAD
P_LUSC
P_PRAD
P_STAD
P_THCA
P_UCEC

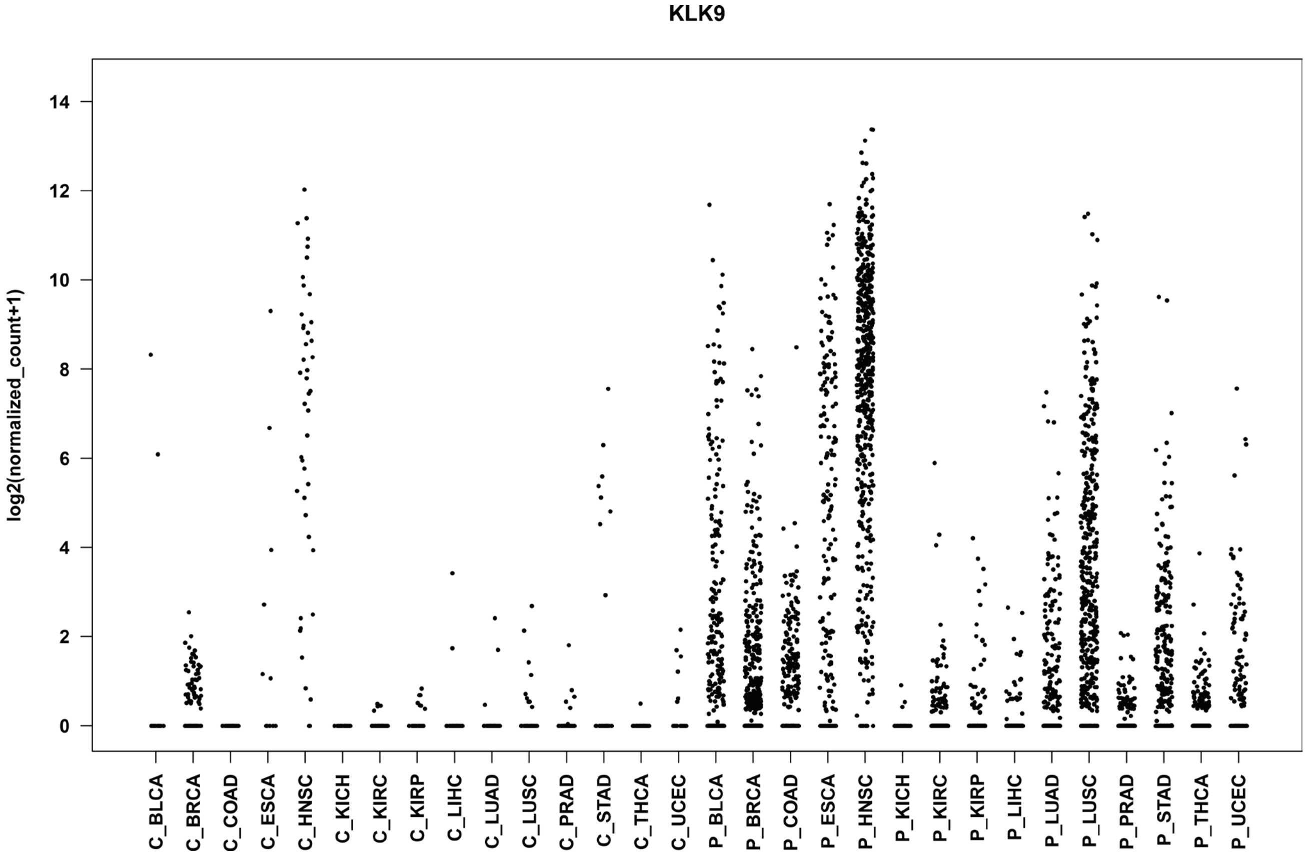
KLK9
log2(normalized_count+1)
14
12
10
8
6
4
2
0
C_BLCA
C_BRCA
C_COAD
C_ESCA
C_HNSC
C_KICH
C_KIRC
C_KIRP
C_LIHC
C_LUAD
C_LUSC
C_PRAD
C_STAD
C_THCA
C_UCEC
P_BLCA
P_BRCA
P_COAD
P_ESCA
P_HNSC
P_KICH
P_KIRC
P_KIRP
P_LIHC
P_LUAD
P_LUSC
P_PRAD
P_STAD
P_THCA
P_UCEC

KLK10

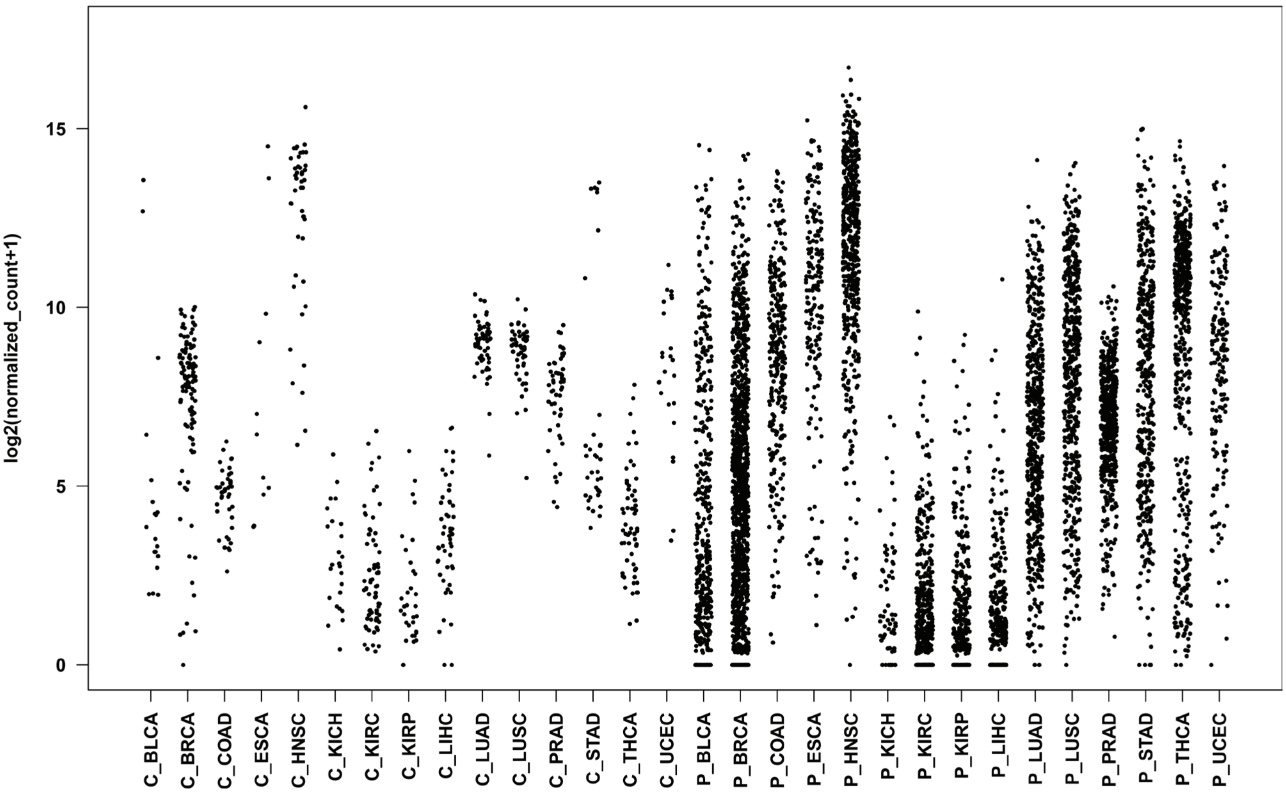

log2(normalized_count+1)
0
5
10
15
C_BLCA
C_BRCA
C_COAD
C_ESCA
C_HNSC
C_KICH
C_KIRC
C_KIRP
C_LIHC
C_LUAD
C_LUSC
C_PRAD
C_STAD
C_THCA
C_UCEC
P_BLCA
P_BRCA
P_COAD
P_ESCA
P_HNSC
P_KICH
P_KIRC
P_KIRP
P_LIHC
P_LUAD
P_LUSC
P_PRAD
P_STAD
P_THCA
P_UCEC

KLK11

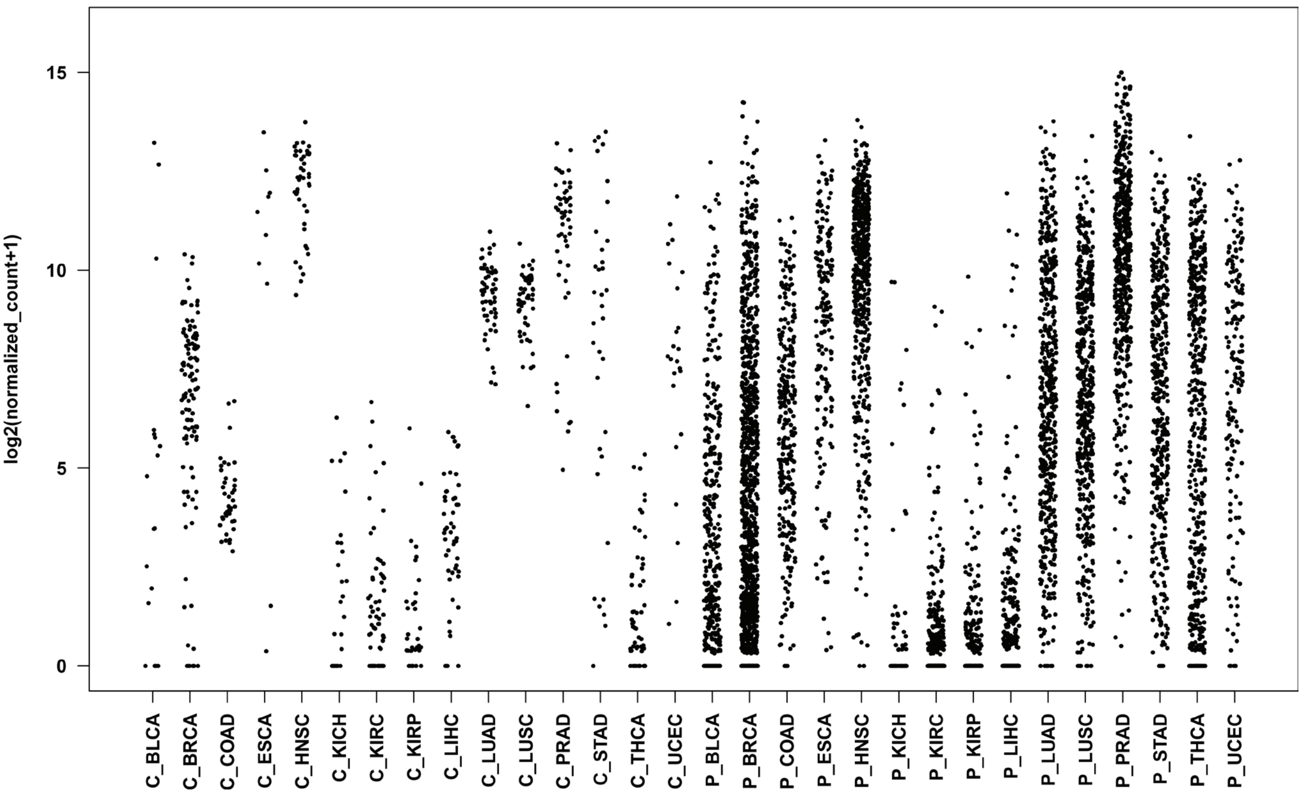

log2(normalized_count+1)
0
5
10
15
C_BLCA
C_BRCA
C_COAD
C_ESCA
C_HNSC
C_KICH
C_KIRC
C_KIRP
C_LIHC
C_LUAD
C_LUSC
C_PRAD
C_STAD
C_THCA
C_UCEC
P_BLCA
P_BRCA
P_COAD
P_ESCA
P_HNSC
P_KICH
P_KIRC
P_KIRP
P_LIHC
P_LUAD
P_LUSC
P_PRAD
P_STAD
P_THCA
P_UCEC

KLK12

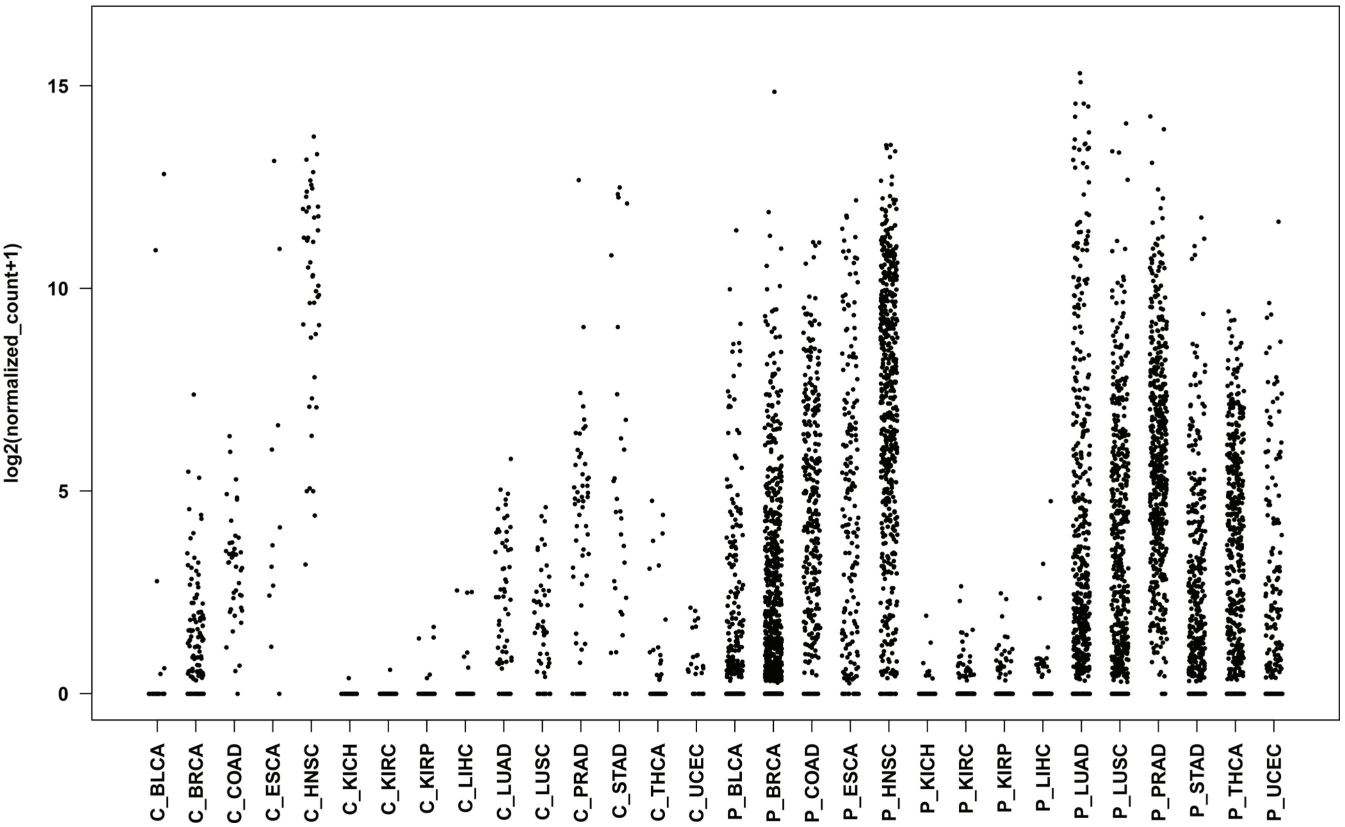
log2(normalized_count+1)
0
5
10
15
C_BLCA
C_BRCA
C_COAD
C_ESCA
C_HNSC
C_KICH
C_KIRC
C_KIRP
C_LIHC
C_LUAD
C_LUSC
C_PRAD
C_STAD
C_THCA
C_UCEC
P_BLCA
P_BRCA
P_COAD
P_ESCA
P_HNSC
P_KICH
P_KIRC
P_KIRP
P_LIHC
P_LUAD
P_LUSC
P_PRAD
P_STAD
P_THCA
P_UCEC

KLK13

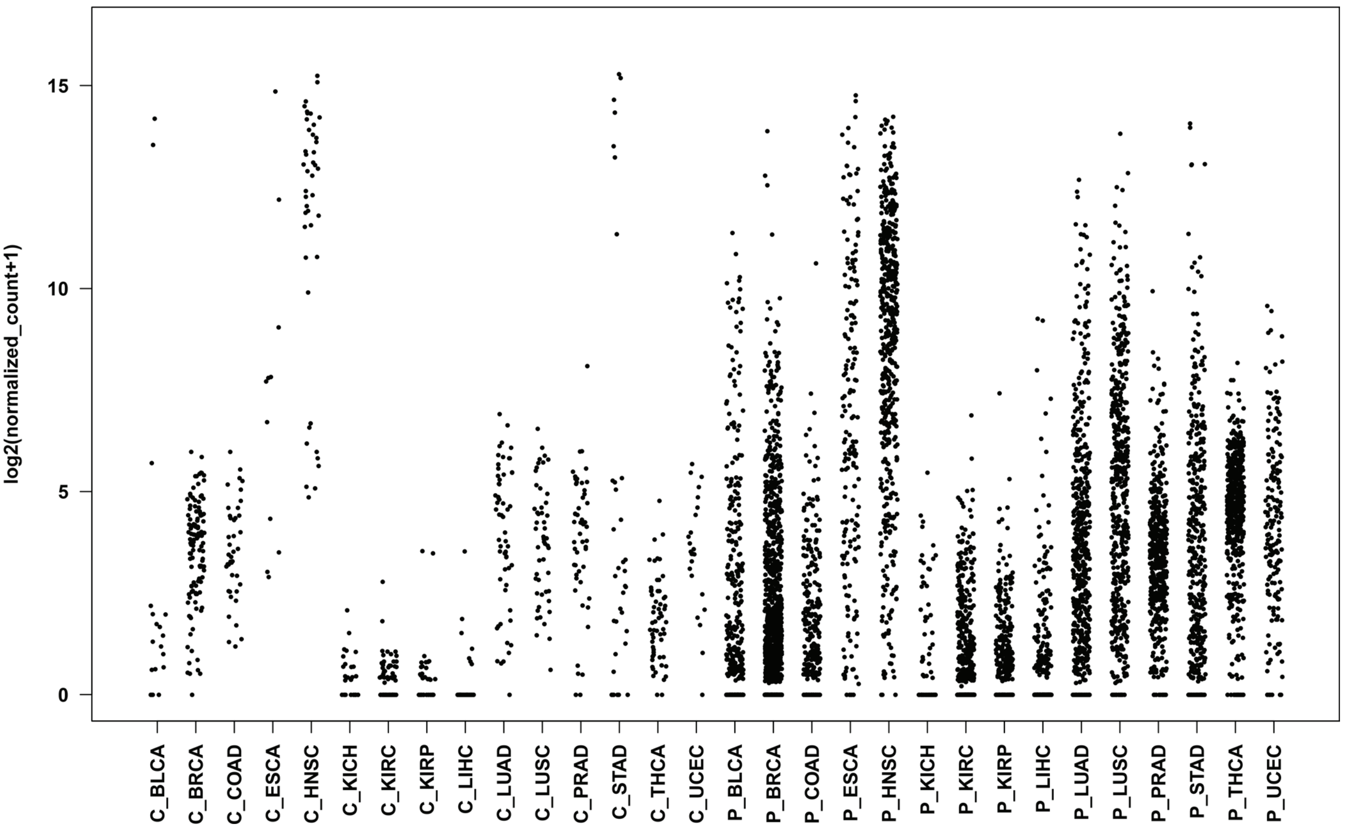
log2(normalized_count+1)
0
5
10
15
C_BLCA
C_BRCA
C_COAD
C_ESCA
C_HNSC
C_KICH
C_KIRC
C_KIRP
C_LIHC
C_LUAD
C_LUSC
C_PRAD
C_STAD
C_THCA
C_UCEC
P_BLCA
P_BRCA
P_COAD
P_ESCA
P_HNSC
P_KICH
P_KIRC
P_KIRP
P_LIHC
P_LUAD
P_LUSC
P_PRAD
P_STAD
P_THCA
P_UCEC

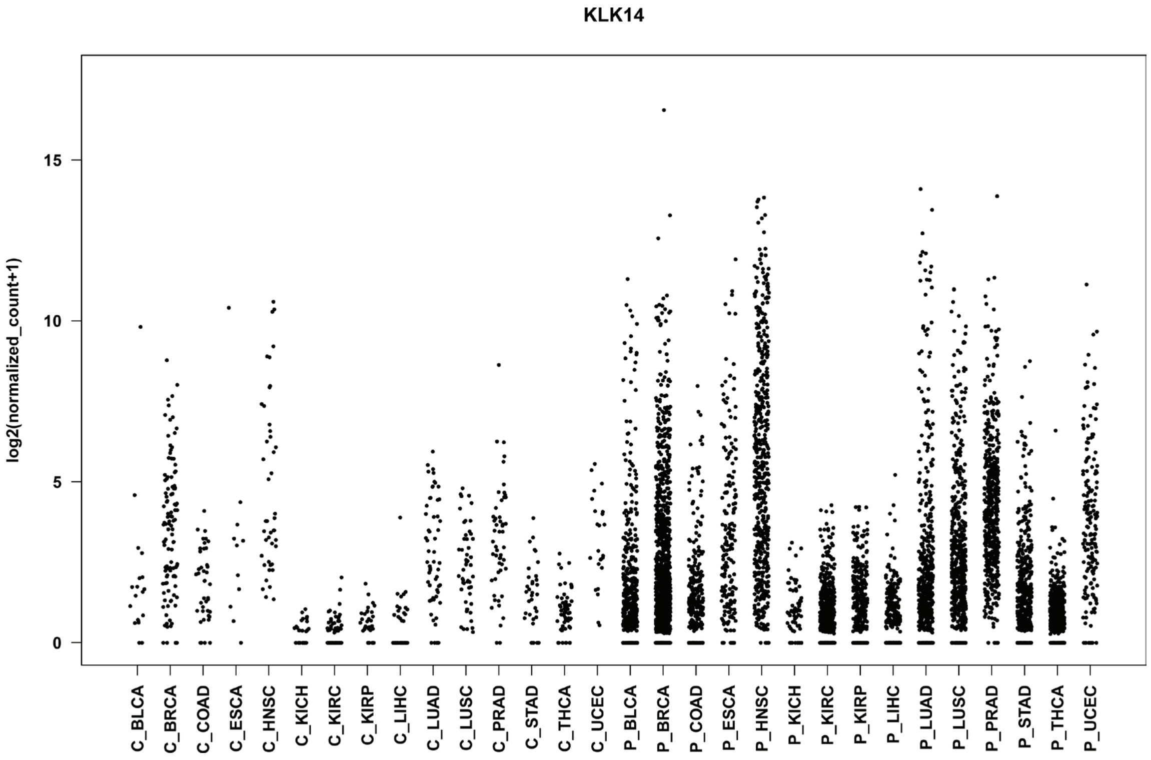

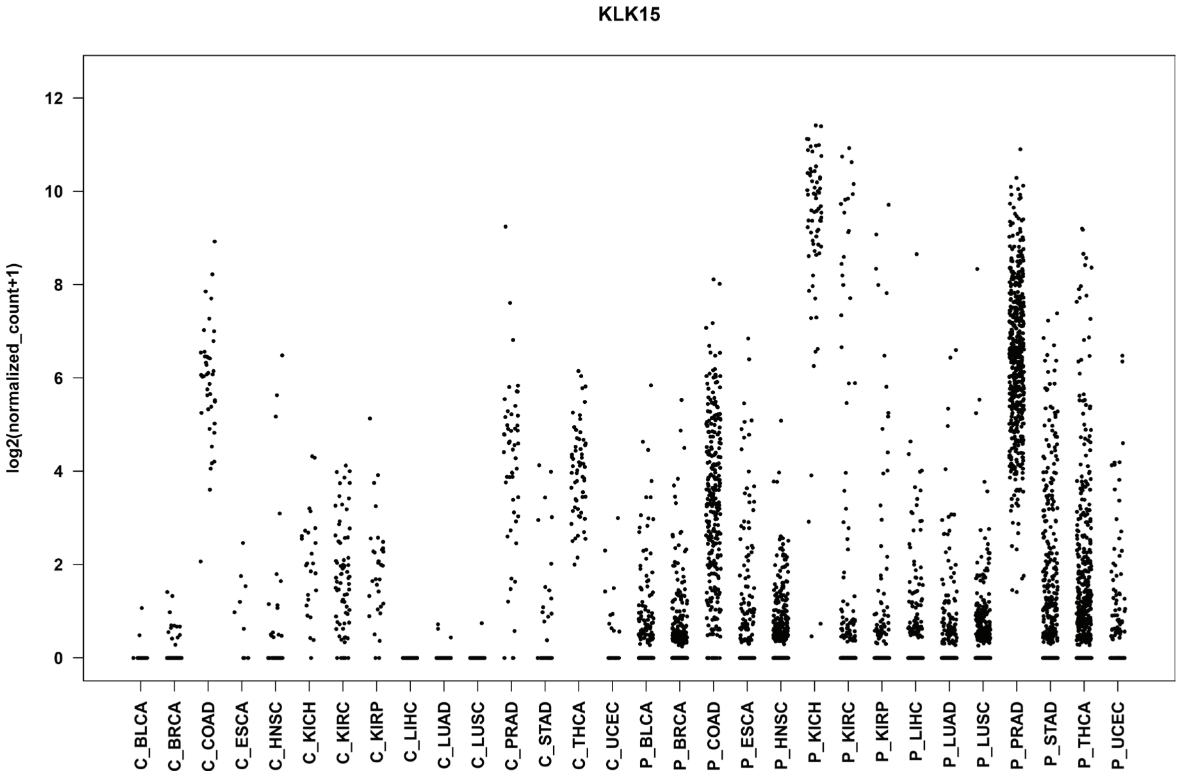

Supplementary Figure 1: The dot-plots showing the distribution of gene expression in cancer patients and adjacent normal for each KLK.
